# Investigation on morphological and molecular fingerprints of penguin brain using label-free optical imaging and spectroscopic techniques

**DOI:** 10.1038/s41598-024-76127-0

**Published:** 2025-02-10

**Authors:** Sunil Bhatt, Ashwani Kumar Verma, Prabir G. Dastidar, Punit Kumar, Pramila Thapa, Tony George Jacob, Tara Sankar Roy, Soumya Iyengar, Senthil Kumaran, Balpreet Singh Ahluwalia, Dalip Singh Mehta

**Affiliations:** 1 Bio-Photonics and Green-Photonics Laboratory, Department of Physics, Indian Institute of Technology Delhi, Hauz-Khas, New Delhi, 110016 India; 2 Polar Sciences Division, Ministry of Earth Sciences, New Delhi, 110003 India; 3 Department of Anatomy, All India Institute of Medical Sciences (AIIMS), Delhi, India; 4 Department of Anatomy, North Delhi Municipal Corporation Medical College and Hindu Rao Hospital, Delhi, India; 5 National Brain Research Centre, Manesar, India; 6 Department of Physics and Technology, UiT The Arctic University of Norway, Tromsø, Norway

**Keywords:** Label-free imaging, Quantitative phase imaging, Autofluorescence spectroscopy, Raman spectroscopy, Penguin brain, Biological techniques, Biophysics, Neuroscience, Climate sciences, Biomarkers, Neurology, Optics and photonics, Physics

## Abstract

The morphology and molecular study of the penguin brain are crucial to define its survival in the extreme conditions of Antarctica. The present study focusses on extracting different optical parameters of the penguin brain using label-free optical imaging and spectroscopic techniques. In label-free optical imaging, we have used quantitative phase imaging, which provides morphological information about the neurons in brain tissue, giving the quantitative phase value of 5 to 20 radians corresponding to the 8 µm tissue section. In label-free spectroscopic techniques, we have used autofluorescence and Raman spectroscopy. Autofluorescence spectroscopy provides molecular information about nicotinamide dinucleotide, flavins, lipofuscins, and porphyrins in the brain’s spectral range of 420 nm to 700 nm. Raman spectroscopy provides multiple peaks associated with different molecules in the brain; among them, few signals are observed at approximately 1305 cm^−1^, 1448 cm^−1^, and 1661 cm^−1^, which correspond to vibrational modes indicative of vibrational features within lipids and protein structures, as well as the presence of amide groups within brain tissue constituents. All these techniques provide the microscopic and molecular fingerprint of the penguin brain, which can be useful for understanding penguin’s anatomical, physiological, and social behavior.

## Introduction

Penguins are flightless birds, one of the world’s oldest surviving species, with skeletal remains dating back to 60 million years^1^. They have an exceptionally well-organized and networked social life. Their cooperative lifestyle in communal rookeries in Antarctica’s harsh environment appears to have had an impact on their physiological and anatomical characteristics. Despite having a primitive brain, penguins demonstrate remarkable anatomical, physiological, and social adaptability to the extreme climatic conditions of Antarctica. Details of the penguin brain are currently unavailable, which would be responsible for their existence in the extreme climatic conditions of Antarctica. The objective of this study is to extract different optical parameters of the penguin brain, and on the basis of these parameters, the ultimate goal is to create a comparative digital brain that can be sustained in a very low-temperature condition in which the penguins live. The main obstacle for studying the brain is its semi-transparent nature, which makes optical techniques less reliable due to low contrast while imaging. In general, the most promising optical techniques used to visualize the tissue structure are bright field (BF), phase contrast (PC), differential interference contrast (DIC), quantitative phase imaging (QPI), and fluorescence microscopy^2–7^. Out of these techniques, BF results in low contrast, PC and DIC provide high contrast images but without any quantitative information, and fluorescence microscopy is a labelled technique where a specific dye is tagged to a specific protein, antibody, or amino acid inside the cells for imaging. Although fluorescence microscopy is used to visualize the dynamics of tissues, cells, and individual internal cell organelles with high contrast, high specificity, and multi-color organelle labelling, it has certain limitations. In labelled imaging techniques, a specific dye is requisite to stain with different proteins to image the specific biomolecule or organelles inside the living cells, which can be bio-hazardous and damage the cells and tissues. In addition, phototoxicity, quenching of fluorescent signals, and photobleaching are the phenomena that limit the effectiveness of labelled imaging techniques^8^.

To overcome the limitations of labelled techniques and to get high-contrast images of the biological samples, label-free techniques are used. Among all optical imaging, in label-free, primarily BF, DIC, and PC techniques are used, which provide qualitative information about the biological cells and tissues, i.e., no quantitative information can be extracted using these techniques. For quantitative information with high-contrast images of the biological samples, one uses QPI, which utilizes the intrinsic contrast of the object to offer superior contrast^9^. Although the QPI techniques provide high-contrast images with quantitative phase information, they are limited to providing microscopic (cellular or subcellular) information about the cells or tissue structures. Thus, other than the optical imaging techniques used to investigate the different optical parameters, label-free optical spectroscopy techniques have also been used, which can provide molecular-level information in biological samples. Two main label-free optical spectroscopy techniques are primarily used: autofluorescence and Raman spectroscopy. Autofluorescence spectroscopy provides global molecular information related to the sample. These molecules have roles in tissue functioning, growth, energy transfer, etc. Meanwhile, owing to unique vibrational spectroscopic specificities, Raman spectroscopy is extensively used to investigate the chemical fingerprint characterization of the vast category of probe analytes and targets molecular structure identification^10^.

Here, we combined label-free optical imaging and spectroscopic techniques to identify different parameters present in the penguin brain. We used label-free optical imaging techniques such as BF, PC, and QPI for the microscopy study and provided information at the cellular level, such as cellular morphology and optical thickness (phase) of the specimen imaged. In addition, for the validation of QPI images, we correlate those images with the standard histopathology/CV (cresyl violet) stained images. CV is a dye that specifically stains the DNA and RNA in neurons and its associated glial cells in the brain tissue samples^11,12^. Secondly, we used autofluorescence and Raman spectroscopy in molecular studies, which provides molecular-specific information and fingerprints about the specimen. To enhance the weak Raman signal, we performed surface-enhanced Raman spectroscopy (SERS) in our study. We have used silver nano-dendrites (AgNDs) as a SERS material for signal amplification in Raman spectroscopy. In the past, several studies have been done on the brain^13–18^; based on those published literature, we have used optical techniques to investigate optical parameters in penguins. This is the first study that will give comprehensive information on the penguin brain and open the research window to study the penguin brain.

## Materials and methods

QPI is a label-free, non-invasive imaging technique used to quantify different parameters, such as morphology, phase, refractive index, height map, dry mass, membrane fluctuation, cell growth, cell motion, etc., about the sample^9,19–22^. A white light phase-shifting interference microscopy (WL-PSIM) system is utilized for the QPI of the brain sample^23–25^. Autofluorescence spectroscopy is another label-free technique that provides molecular information in which a particular excitation wavelength of light is made to illuminate the sample; then endogenous fluorophores, such as proteins, lipids, antibodies, amino acids, etc., absorb the incident light and emit a particular low energy light in emission^26,27^. The light source with an excitation wavelength of 405 nm was used for autofluorescence spectroscopy. A Renishaw Raman microscope with incident light having a central wavelength of 785 nm was used for Raman spectroscopy. In SERS, due to the extreme confinement of the local fields at the sharp tips and within the intra-branch and inter-branch nanogaps of AgNDs, it offers highly dense three-dimensional (3D) SERS “hot spots” and consequentially enables the prominent signal enhancements for the differentiated identification of constituting tissue bio-analytes.

All data acquisition and processing were performed using standard optical systems described in Supplementary Figs. 2 and 3 and Supplementary Methods S1–S3.

### Sample procurement and preparations

#### Tissue sample preparation

**Fig. 1:**
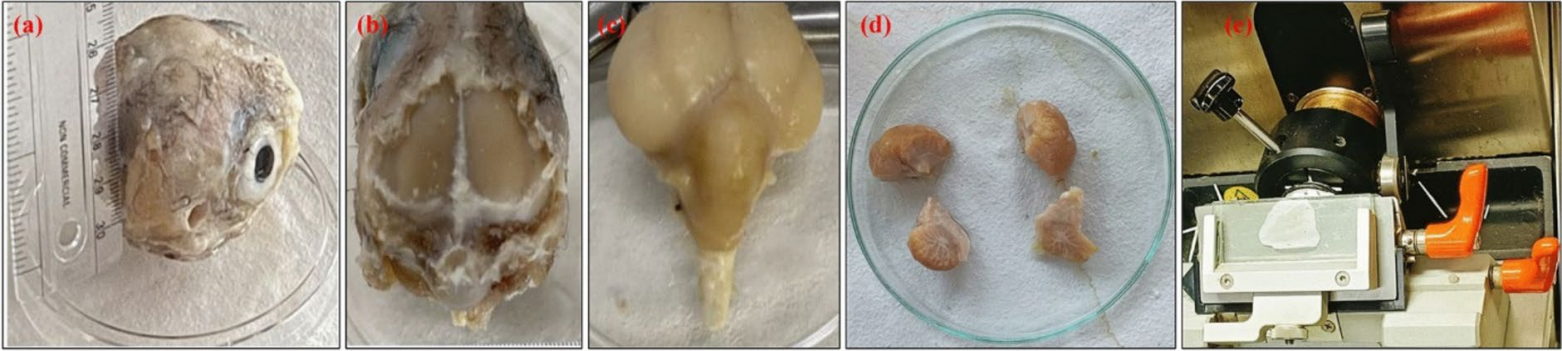
Illustrating different stages of the penguin brain dissection. (**a**) penguin head (**b**) brain in partially dissected skull (**c**) penguin brain after dissection (**d**) subdivided brain tissue for sectioning (**e**) tissue sample on a glass slide in the microtome.

Penguin heads were collected from penguin carcasses (natural death) on Svennar Island, Antarctica (S 69°08′12″, E 76°44′45″), during the 39th Indian expedition to Antarctica. The penguin carcass was decapitated, and the head was fixed by immersion in chilled buffered paraformaldehyde (made in 0.1 M phosphate buffer (PB), pH 7.4) immediately after collecting the heads of the penguins on the Island. It was then cleaned in distilled water and kept in the fixative solution at 4 °C, which was changed every seven days for two months. It ensured gradual penetration of the fixatives to the sample, fixed the tissues without deterioration, and finally brought them to India for study, as shown in Fig. 1a. The brain was carefully dissected out from the bony skull, as shown in Fig. 1b,c, and divided into four parts so that it perfectly fits on the microtome for sectioning, as shown in Fig. 1d. The dissected parts were embedded in a cryomedium for cryoprotection. In cryo-sectioning, the fixed brain was washed in PB and cryoprotected in 30% sucrose (in 0.1 M phosphate buffer, pH 7.4). The brain was mounted in a cryomedium (Cryomatrix, Thermo Scientific, UK) and sectioned coronally on a cryotome (Microm International GmbH, Germany) to obtain 8 µm thin sections, as shown in Fig. 1e. Alternatively, the successive sections were used to prepare the samples on the silicon wafer and the glass slides. The samples over the wafers were used for the QPI imaging, while the samples over the glass slides were stained with cresyl violet (CV) (1%) for visualizing the neurons inside the brain. A thick section of tissues was used to prepare the sample over the glass slide to perform AF and Raman spectroscopy. Before performing the AF and Raman spectroscopy, the fixative was removed by washing the brain three times in 0.1 M phosphate buffer, pH 7.4, for 10 min each. For all the experimentation and to perform repeatability in results, we used multiple sections from two different penguin brains derived from two penguin heads.

#### Ethical clearance

Protocols prescribed by the Antarctica Treaty Consultative Meeting (ATCM) Resolution 4 (2019)^28^ and Annex II to the protocol on Environmental Protection to the Antarctic Treaty^29^ were followed during our research studies in Antarctica. Only the carcasses of penguins that were found naturally dead were used in this observational study. All the experiments were conducted ex vivo and followed all the ARRIVE guidelines. The penguin heads were preserved by immersion in buffered paraformaldehyde prior to extracting the brain from them. Further preparation of the samples for microscopy, etc., was carried out at the All-India Institute of Medical Sciences (AIIMS), New Delhi. Brain tissue sections taken on a glass slide and covered with a coverslip were brought to the Indian Institute of Technology Delhi (IITD) for phase microscopy and spectroscopy. Further, we confirm that all methods and experimentation were performed in accordance with the relevant guidelines and regulations for using vertebrates for research studies. We have approved ethical clearance for the study from the Institutional Animal Ethics Committee AIIMS with certificate number 484/IAEC-1/2024.

#### Material specifications to prepare silver nano dendrites

Analytical grade silver nitrate (AgNO_3_, 99.9%, SRL), l-ascorbic acid (L-AA, 99.7%, SRL), and polyvinylpyrrolidone powder (PVP, MW ~ 55,000, 99%, Sigma Aldrich) were used as received otherwise stated. Ethanol, acetone, and deionized water (DI water) were used as a solvent in all experiments.

#### Synthesis of silver nano dendrites for surface-enhanced Raman spectroscopy

As previously reported, the optimized dendritic-shaped multi-branched silver nanostructures (AgNDs) were synthesized using a room-temperature, single-step chemical reduction synthesis^30,31^. In brief, the initial colorless solution of AgNO_3_ (5 ml, 1 mM) immediately (~ 5 s) turned to light grey color after the drop-wise addition of aqueous L-AA (0.25 ml, 500 mM) solution, thus indicating the synthesis of primary silver nanoparticles. Owing to its selective surface interaction with high-energy crystal facts, drop-wise added PVP (0.2 ml, 10 mM) acts as a shape-directing agent by controlling the growth of the nanostructures at the initial stages and then further proceeded by the stabilization of the branched morphology of the final nanostructures. The branching in the morphology of the nanostructures is critically governed by the rapidness of the L-AA-induced chemical reduction of the silver ions. Therefore, the over-growth and aggregation of the as-synthesized AgNDs were prevented by isolating them at the early stage of reaction via centrifugation at ~ 6000 rpm for a 20-min reaction time.

## Result and discussion

To investigate the morphological and molecular fingerprints of the penguin brain, we categorized our study into two sections: microscopic and molecular information about the penguin brain.

### Microscopic information about the penguin brains

We performed this study to visualize the morphology and study the cellular and subcellular information related to the brain tissue sample in label-free mode. The BF imaging and the PC of the brain tissue sample are shown in Supplementary Fig. S1a–c and Supplementary Fig. S1d and d_1_. Although the PC provides high-contrast images, it provides only qualitative visualization. We utilized QPI in our study to get the high contrast morphological and quantitative phase information of the cellular structures inside the brain tissue, such as neurons, blood vessels, blood cells, etc., without any external dye tagging or specific sample preparation.

**Fig. 2:**
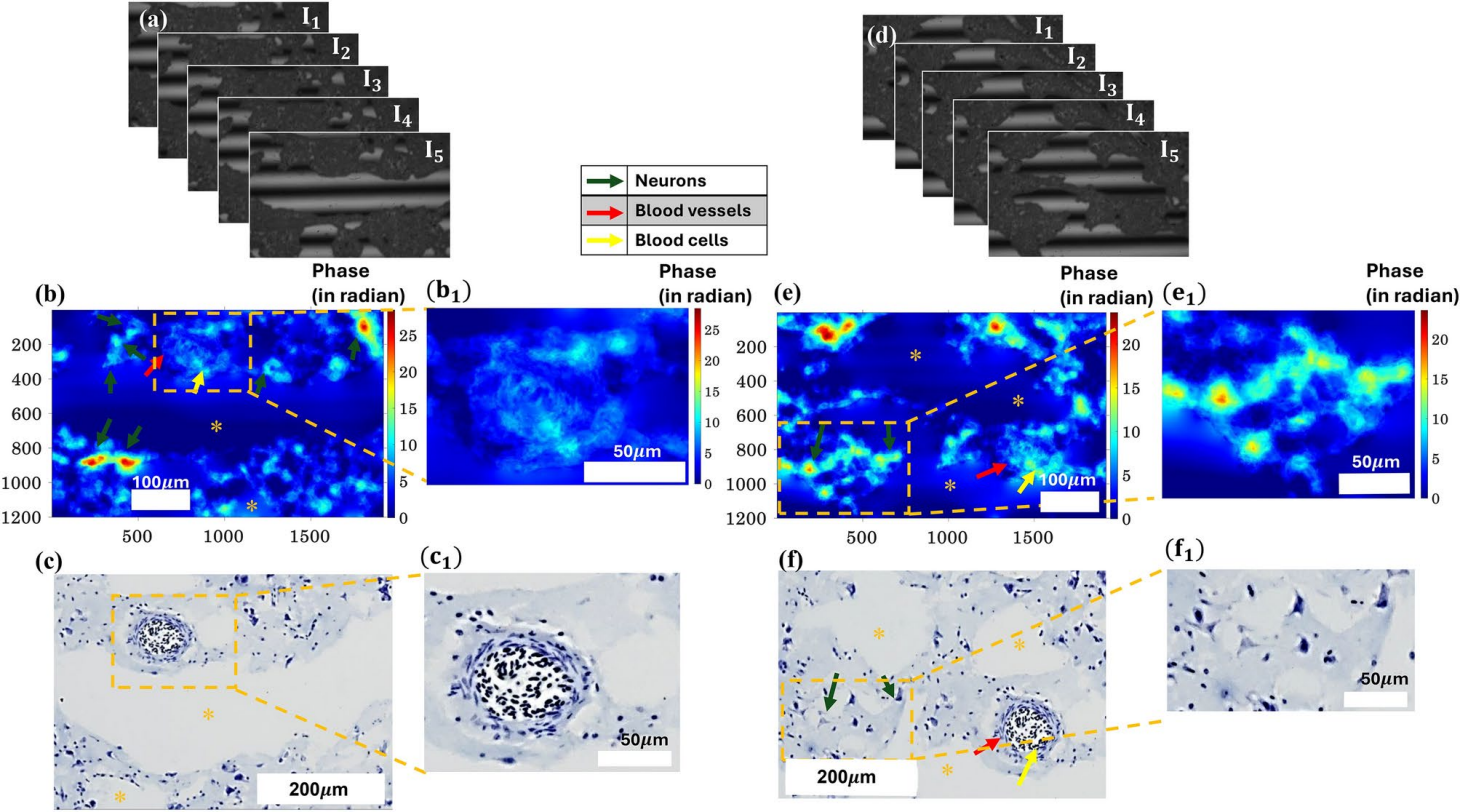
Quantitative phase imaging of penguin brain tissue sample to quantify the morphological information about the cellular structures inside the tissue and its correlation with CV-stained images. (**a**,**d**) are the five phase-shifted interferometric images of the penguin brain tissue. (**b**,**e**) are the corresponding reconstructed phase maps using the phase shifting algorithm respectively. (**b**_**1**_,**e**_**1**_) are the cropped image of region of interest (ROI), specified in yellow rectangular box in (b, e), respectively, showing the phase map of cells inside the brain tissue. (**c**,**f**) are the CV-stained images of the respective brain tissues for the correlation and identification of cells inside the tissues, and (**c**_**1**_,**f**_**1**_) are the cropped ROI, specified in a yellow rectangular box in (**c,f**), respectively. The yellow color (*) represents the artifacts of freezing (irregular spaces).

Figure 2 shows the quantitative phase of the penguin brain sample using the WL-PSIM system and its correlation with CV-stained images for the validation of biological constituents. A detailed description of the experimental setup and working principle of the WL-PSIM system for the QPI is shown in Supplementary Fig. S2 and Supplementary Methods S1, respectively. We have used two different microscope objectives (MO’s) to acquire the datasets. For the phase-shifted interferometric data acquisition, we used a 20X/0.44 Mirau MO lens, while for the CV-stained histopathological imaging for the correlation, we used a 10X/0.44 Plan MO. The reason for choosing two different MO’s is due to the unavailability of the same MO’s for both experimentations since 20X Mirau cannot be used for the brightfield imaging of CV-stained samples. Further, the data acquisition is carried out in two different institutes and optical systems: All India Institute of Medical Sciences (AIIMS) India and Indian Institute of Technology Delhi (IITD).

Figure 2a,d shows the five equal phase-shifted interferograms of the brain tissue acquired using the WL-PSIM system. Using the five-phase-shifting algorithm, we reconstructed the corresponding phase map of the tissues. Figure 2b,e represents the phase map of the brain tissue, which provides cellular and subcellular morphological information about different structures such as neurons, blood vessels, and blood cells. Figure 2b1 shows the ROI, where the blood vessels and blood cells are situated inside the brain tissue sample, while Fig. 2e1 shows the ROI, where neurons are presented. The phase variation in the cells is represented by the colormap at the right corner of Fig. 2. The quantitative phase for the 8um tissue section ranges from 5 to 20 radians for the neurons in the brain, whereas for the blood cells and blood vessels are ranging in between 5 to 12 radians. For the validation of the neurons and other cellular structures inside the brain in label-free mode, i.e., QPI, we used the subsequent sections of tissue for the CV-staining, i.e., cellular-specific labelled technique, shown in Fig. 2c,f, respectively. Figure 2c1,f1 are ROI’s representing one-to-one correlation with the Fig. 2b1,e1, respectively. The one-to-one correlation between Fig. 2b with c and Fig. 2e with f signifies that the label-free techniques can provide high contrast morphological information in addition to the quantitative phase, i.e., coupled information of the refractive index and the thickness inside the brain tissue. The high scattering of the brain tissue hinders the achievement of diffraction-limited resolution in the QPI images; nevertheless, this initial work demonstrates the feasibility of QPI on brain tissue sections of the penguin brain.

### Autofluorescence spectroscopy of penguin brain tissue for molecular information

However, QPI images present label-free high-contrast images of different biological constituents inside the tissue structure, which represents the morphological information with the quantitative phase. There are several biological phenomena whose progression occurs at the molecular level, such as cancer, brain tumor, etc. Investigating molecular-level information helps to understand the molecular insights of the brain, which ultimately can help identify abnormalities inside the tissue structure. Thus, more insight into the brain tissue constituents can be found using autofluorescence and Raman spectroscopy, which provide the biomarkers present in the tissue and are the molecular fingerprints for the tissue. In tissue, different endogenous fluorophores, such as NADH, FAD, proteins, lipids, collagens, etc., are present that are autofluorescence. The motive of the study is to find out the different autofluorescence spectra from the brain tissue.

**Fig. 3:**
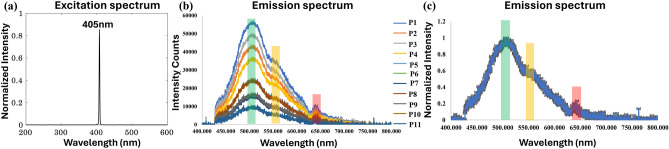
Autofluorescence spectroscopy of penguin brain at excitation wavelength 405 nm (**a**) 405 nm source excitation spectra. (**b**) AF emission spectrum of a total of 11 positions of the penguin brain with intensity variation. (**c**) AF spectra of the average of (**b**) with the standard deviation. The highlighted, green-shaded region in the spectra consistently manifests a peak around ~ 510 nm, corresponding to NADH and FAD crosslinks. The highlighted, red-shaded region within the spectra depicts peak maxima approximately at ~ 656 nm, indicative of porphyrins. The highlighted, yellow-shaded region in a range of 550 nm to 600 nm is an indicator of lipofuscins.

Figure 3a shows the excitation spectra of light sources used for autofluorescence in penguin brain tissue. The detailed description and experimental setup for autofluorescence spectroscopy are shown in Supplementary Fig. S3 and Supplementary Methods S2. Autofluorescence in the visible region results mainly from the intrinsic chromophores such as nicotinamide adenine dinucleotide phosphate (NADP), flavin in intracellular compartments, collagen, elastin, and NADH, etc.^26,32,33^. For 405 nm excitation light, we have used a 420 nm long pass filter to cut off the excitation light from the emission spectra. The autofluorescence (AF) spectra of the penguin brain (PB) were analysed following excitation at 405 nm, encompassing a spectral range from 425 to 800 nm. This range captures the emission signals of various endogenous fluorophores. Specifically, the spectral band spanning from 450 to 550 nm revealed prominent features attributed to NADH-FAD crosslinks, indicative of the presence of these key fluorophores^34^. Figure 3b displays the AF spectra obtained from 11 distinct positions within the penguin brain, illustrating the intensity distribution across these locations while maintaining consistent spectral characteristics. Subsequently, Fig. 3c presents the average spectra derived from the data in Fig. 3b, accompanied by standard deviation indicators. The central blue region signifies the mean spectral profile, while the surrounding grey lines represent the variation observed across multiple recorded spectra. Highlighted within both Fig. 3b and c, the green-shaded region in the spectra consistently manifests a peak around ~ 510 nm, corresponding to NADH and FAD crosslinks^34^. These crosslinks, serving as primary metabolic coenzymes within biological specimens, demonstrate uniform presence across all sampled positions^34^. In addition to NADH and FAD crosslinks, the extracellular matrix proteins such as collagen, elastin, and laminin play pivotal roles in furnishing structural integrity and establishing specialized microenvironments conducive to intricate neuronal and glial interplay within the central nervous system (CNS) referring to the peaks ranging from 420 to 510 nm in Fig. 3b^26,35^. Furthermore, the red-shaded region within the spectra depicts peak maxima approximately at ~ 656 nm, indicative of porphyrins. This spectral feature suggests the presence of blood within the brain, as porphyrins constitute a principal component of hemoglobin^34^. In the wavelength range of 550 nm to 600 nm, lipofuscins are evident, as depicted in Fig. 3b and c by the yellow-shaded region. One notable phenomenon observed in the aging brain involves the conspicuous buildup of lipofuscin aggregates alongside neuromelanin pigments^36^.

In addition, the repeatability of the current methodology was investigated by collecting the AF spectra from the 10 different locations of other penguin brain tissue specimens, and the corresponding spectra are shown in Supplementary Fig. S4. Table 1 lists the probable endogenous fluorophores by evaluating experimentally obtained AF spectra along 11 different positions of the penguin brain with their tentative biological constituents.

**Table 1:** Endogenous fluorophores exhibiting autofluorescence spectra along 11 different positions at an excitation wavelength of 405 nm on the penguin brain^26,32,33,35,37–39^.

Endogenous fluorophore	Excitation wavelength	Emission wavelength	Biological constituents
NADH	405 nm (less absorption)	420–500 nm	Coenzyme central to metabolism
Elastin and Collagen	405 nm (350–420 nm)	420–510 nm	Extracellular fibrous proteins
Protoporphyrin IX and Porphyrin derivatives	405 nm	630–700 nm/(630, 660, and 690 nm)	Protein prosthetic group such as hemoglobin
Lipofuscins/lipofuscin like-lipopigments/ceroids	405 nm (400–500 nm)	480–700 nm	Proteins, lipids, retinoids
FAD	405 nm (less absorption)	540 nm (520-560 nm)	Redox-active coenzyme

### Raman spectroscopy for molecular fingerprints

**Fig. 4:**
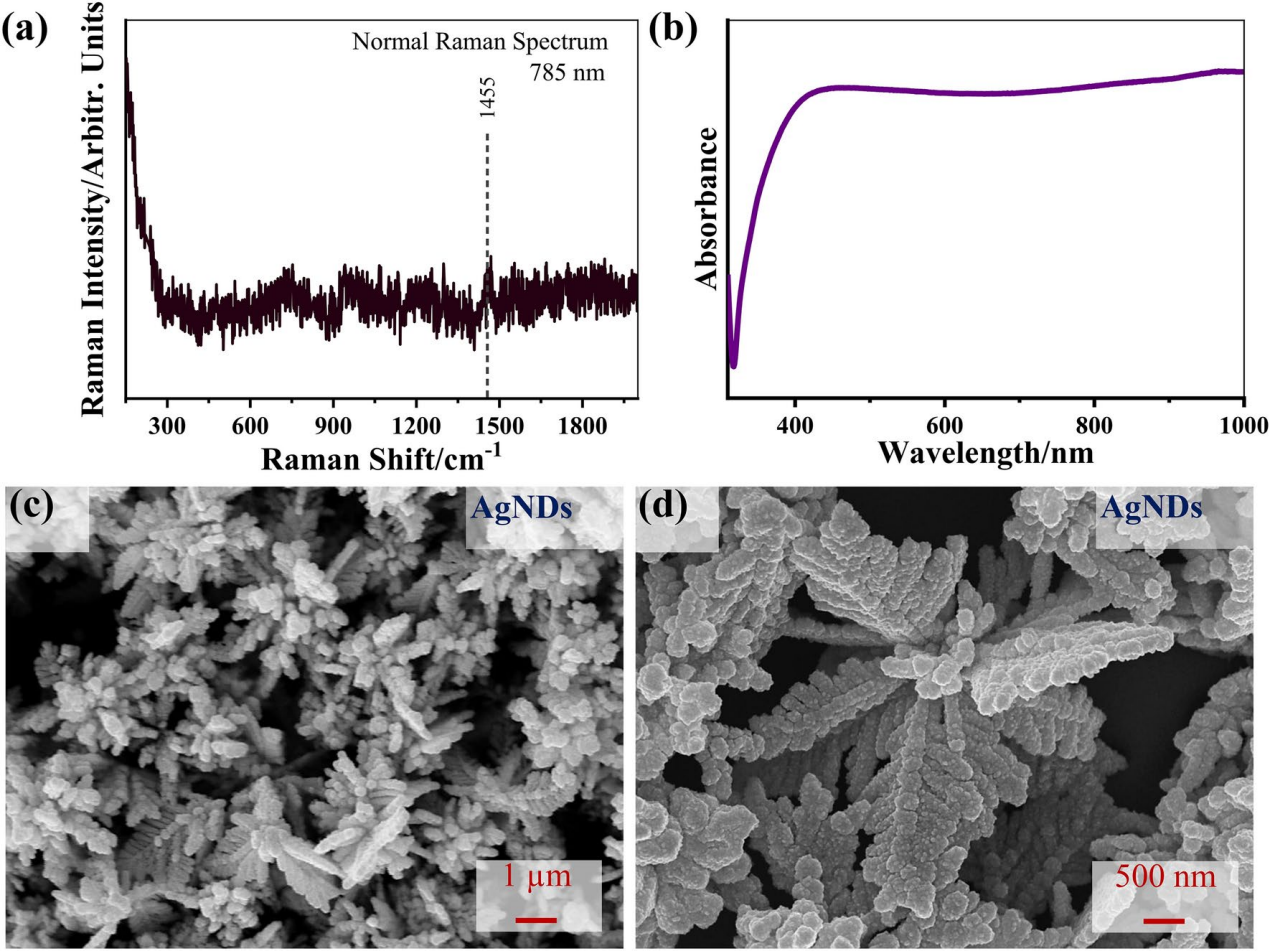
(**a**) Normal Raman spectra of the Penguin brain tissue under NIR (785 nm) excitation wavelength, (**b**) UV–Vis absorption spectrum of colloidal multi-branched silver nanodendrites (AgNDs) aqueous solution, and (**c**,**d**) FESEM images of the respective AgNDs.

In order to study the molecular fingerprints of constituents of the penguin brain tissue, the normal Raman and SERS spectra were acquired with NIR (785 nm) excitation wavelength. With the application of NIR excitation, plasmonic nanostructures serve as fluorescence quenchers as well as effectively resolve the self-absorption of tissue constituents such as hemoglobin and water, depicting the low absorption within the first biological window (700 to 1000 nm). The subsequently suppressed absorption also provides the in-depth analysis of tissue constituents by enabling deeper penetration in the tissue as well as promoting in vivo/ex vivo and non-invasive characterization through negligible photochemical damage of the tissue specimens under the exposure of less-energetic incident NIR photons^40^. Therefore, the current work utilizes the NIR excitation wavelength to study the whole spectral features of penguin brain tissue specimens benefitting from the quenched fluorescence and reduced self-absorption. The experimental configuration and details for Raman spectroscopy are discussed in Supplementary Methods S3. As seen from Fig. 4a, the conventional Raman spectroscopy of penguin brain tissues does not depict any visible information and thus would benefit from SERS for signal amplification. However, a weak peak has been obtained ~ 1455 cm^−1^ (Fig. 4a) assigned to the “δ(CN) bending, δ(CH_3_) out-of-phase deformation of lipid and protein”, which confirms the presence of lipids in the penguin brain tissue specimens (Table 2).

**Table 2:** SERS peak positions and the tentative vibrational assignments of the penguin brain tissue specimens under NIR (785 nm) excitation wavelength^16,17,44–47,51–56^.

Peak position (cm^−1^)	Assignments
547	Amino acids (tryptophan) combined with nucleobases DNA and RNA (cytosine and guanine)
623	C–C twisting mode of phenylalanine
642	C–C twisting mode of tyrosine
661	C–S stretching mode of cystine
704	CH_2_ rocking coupled with symmetric breathing of l-tyrosine
719	Symmetric choline C–N stretch (membrane phospholipid head) coupled with ring breathing mode of DNA/RNA nucleobases adenine
744	CH_2_ rocking coupled with symmetric breathing of tryptophan, mitochondria, and cytochrome c
880	Secondary amino acid proline
964	Hydroxyapatite (PO_4_^3−^ symmetric stretching)/carotenoid/cholesterol
978	C-H out-of-plane deformation coupled with C–C asymmetric stretching of Deoxygenated cells, cytochrome c, and porphyrin
1003	Symmetric C–C aromatic ring breathing mode of phenylalanine and collagen IV, and I
1067	C–C stretching coupled with C–C skeletal stretching, PO_2_ symmetric stretching of nucleic acid, protein, phospholipid, glycogen, and collagen IV
1088	
1131	C–N stretching in proteins
1158	C–C stretching of carotenoids coupled with C–N stretching of proteins
1179	C-H in-plane bending of hemoglobin, tyrosine, and flavin
1273	In-plane deformation of N–H, C–N stretching
1305	CH_3_ in-plane deformation of amide III
1351	CH_3−_ (C=O) tryptophan, mitochondria, cytochrome c
1384	CH_3_ in-phase deformation, methyl symmetric bending of thymine, adenine, and guanine of DNA
1399	CH_3_ bending due to methyl bond in the membrane
1448	δ(CN) bending, δ(CH)_3_ out-of-phase deformation of lipid and protein
1556	C=C tryptophan, porphyrin
1622	C=C stretching mode of tyrosine and tryptophan
1661	Amide I (proteins with b-sheet conformational structures), ν(C=O), and collagen I, IV

The SERS detection requires the proper implementation of plasmonically active nanostructures to obtain significantly enhanced Raman signals. Owing to superior SERS activity, highly anisotropic multi-branched dendritic-shaped silver nanostructures are implemented in the present study. The localized surface plasmon resonance (LSPR)-induced field localization plays a vital role in overall Raman intensity amplification by efficiently supporting the dominant electromagnetic enhancement mechanism. The LSPR is mainly attributed to the collective oscillation of the conduction electron gas on the metallic features caused by the light irradiation. The electromagnetic enhancement critically depends on the structural morphology and compositional nature as well as on the relative arrangement or distribution of nanostructures over the substrates, supporting near-field interactions among the nanostructures^41^. The controlled nano-scale inter-particle spacings substantially impact the optical response through plasmonic couplings, thus consecutively leading to the stronger localization of the incident field, producing highly dense “hot-spots” with inter/intra-particle gaps. The anisotropically engineered nanostructures decorated with multiple sharp primary and secondary branches depicting nano-scale intra-branch gaps have proven their superior practicability for sensitive molecular identification applications^42,43^. In this work, we systematically explored the comparative signal enhancement capabilities of the two different configurations for the implementation of plasmonic dendritic-shaped silver nanostructures under NIR (785 nm) excitation wavelength.

**Fig. 5:**
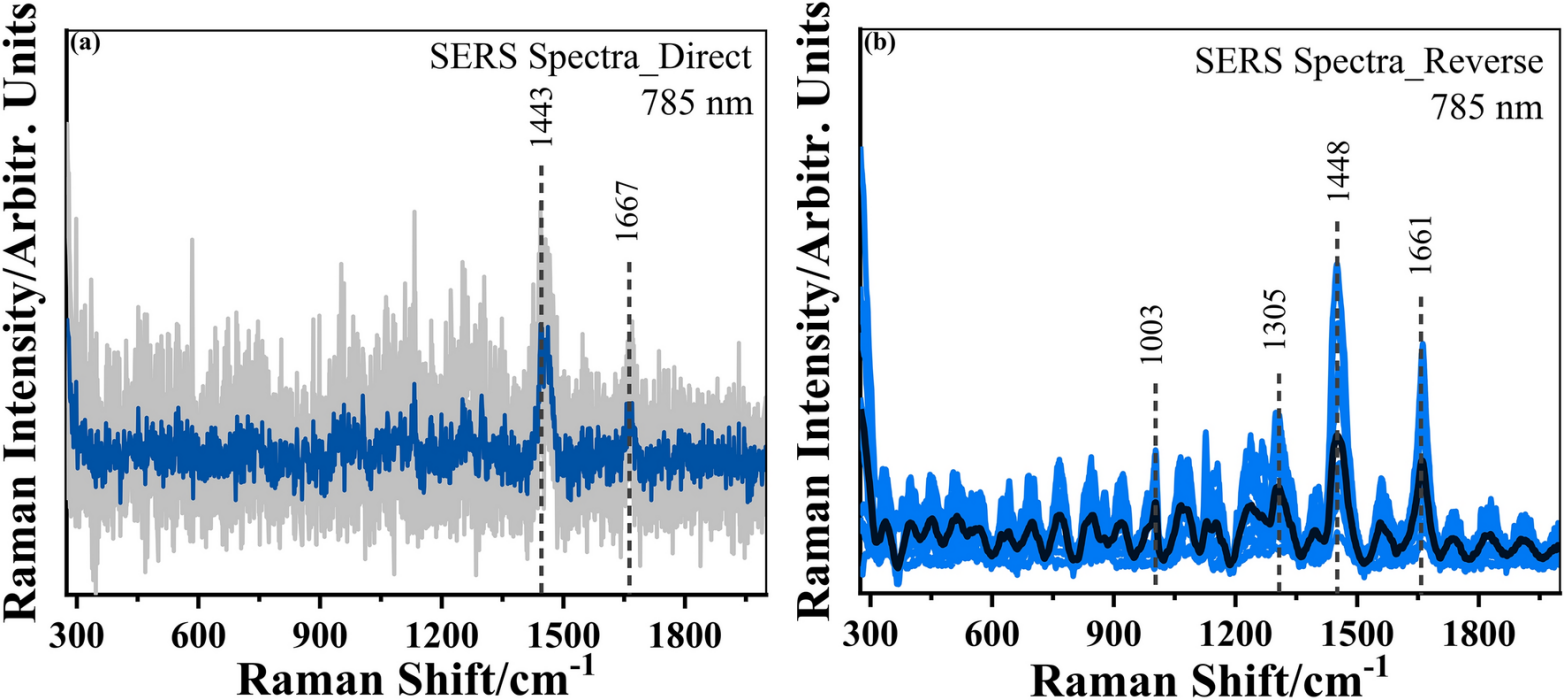
The collected average SERS spectra of penguin brain by (**a**) direct depositing the tissue specimens over plasmonic silver nanodendrites at 5 different positions and (**b**) reversely drop-coating the colloidal AgNDs solution on the top of the tissue specimens, under NIR (785 nm) excitation wavelength at 15 different positions.

Figure 4b–d detail the UV–Vis absorption spectrum and morphological analysis of the as-synthesized plasmonic dendritic-shaped branched silver nanostructures (AgNDs). For AgNDs, the optimized molar ratio of involved chemical reagents AgNO_3_:L-AA (R_1_) and AgNO_3_:PVP (R_2_) are 0.04 and 2.5, respectively, and the corresponding FESEM images depict the well-defined multi-branched morphology. The selective surface interaction of PVP with the silver crystal facets initiates the preferential growth along {111} crystal planes, leading to the emergence of primary and secondary branches^30,31^. The hybridization of long-wavelength plasmons associated with individual tips to the short-wavelength plasmons associated with the core produces a broad extinction spectrum from 350 to 1000 nm due to the associated structural heterogeneity, as clearly revealed in Fig. 4b. The extended morphological analysis reveals the decoration of the central core with primary branches constituted with sharp secondary branches with an average arm length of 2 μm separated by nano-scale intra-branch gaps (Fig. 4c and d). The micro/nano-scale sharp branches, along with nano-sized intra-branch gaps, provide strong electric-field localization that is beneficial for SERS-based detection applications. Raman spectrum of bare AgNDs deposited over cleaned silicon substrates under 785 nm laser excitation wavelength is shown in Supplementary Fig. S5. Figure 5a comparatively represents average SERS spectra of penguin brain tissues deposited over plasmonically active silver nanodendrites substrates. The corresponding spectra depict the absence of much clear information related to the constituents of the penguin brain tissue specimens. We have observed that the subsequent Raman spectra revealed very little information on the fingerprints of brain tissue with few weak Raman modes at ~ 1443 and 1667 cm^−1^, due to the absence of clear spectral peaks and poor signal-to-noise ratio^16,44,45^. Therefore, owing to its strong distance-dependent near-field plasmonic activity of the fabricated nanostructures, the currently employed strategy utilizing the penguin brain tissues deposited over plasmonic nanostructures is unable to properly amplify the Raman signals and thus restricts the applicability of the conventional SERS approach^46^.

The mentioned experimental constraints related to the limited near-field plasmonic activity of nanostructures can be efficiently overcome by adopting a reverse methodology through depositing an aliquot (~ 10 µl) of concentrated ethanolic solution of AgNDs over the upper surface of the penguin brain tissue specimens^45^. Then, the reproducible NIR-SERS spectra were directly collected from the multiple locations of immediately dried dendritic nanostructures deposited on the tissue specimen surface to analyse the overall molecular information. Anisotropically engineered nanostructures have been explored as efficient platforms providing multiple highly dense intrinsic plasmonic “hot spot” rich regions without the aggregation of nanoparticles. Owing to their distinctive spectral features, the constitution of tissue specimens in the form of peptides, amino acids, proteins, and amide linkages between amino acids requires the proper analysis of the whole Raman spectrum, specifically within the 300–1700 cm^−1^ spectral regime. In contrast, the presence of amino acids such as tryptophan, tyrosine, and phenylalanine and the amide linkages between amino acids in the form of amide I and amide III are usually employed to confirm the predominated presence of proteins^16,17,44–47^. The experimentally obtained spectra in Fig. 5b from 15 different positions of brain tissue clearly reveal the spectral analysis of the SERS spectra exhibiting the prominent Raman band regions at ~ 700 cm^−1^, 850–900 cm^−1^, 1000 cm^−1^, 1030–1150 cm^−1^, 1230–1350 cm^−1^, 1420–1460 cm^−1^, and 1610–1690 cm^−1^, thus showing the distinctive compositional variations of the penguin brain tissue specimens. However, one can clearly observe that the respective SERS spectra from the identical samples differ significantly from the previous normal Raman and direct SERS spectra in terms of the relative intensity and signal-to-noise ratio of the Raman modes. The spectral overlap of the 785 nm laser excitation line with the broad plasmon response of AgNDs resulted in stronger LSPR and, accordingly, sufficiently high-intensity enhancement of the Raman modes due to the generation of highly dense intrinsic hot-spot-rich regions. Notably, the SERS spectra resulted due to the subsequent interaction of the penguin brain tissue constituents with the active metallic sites of large-area AgNDs and then correspondingly exhibit many intense Raman bands in the low (below 1000 cm^−1^) as well as high (above 1000 cm^−1^) wavenumber spectral regimes. The close similarities of the SERS spectra from different brain locations are possibly attributed to the close proximity or the specific distance-dependent interaction of constituting brain molecules having a particular affinity with the silver nanodendrites surface^45^. In addition, the reliability of the current reverse methodology was investigated by collecting the SERS spectra from the 10 different locations of other penguin brain tissue specimens. The corresponding spectra are shown in Supplementary Fig. S6. Table 2 lists the experimentally obtained Raman modes along with their tentative vibrational assignments representing the tissue’s constituents. Importantly, two penguin brain specimens exhibited nearly similar vibrational features without any noticeable spectral difference. The spectral differences and similarities of the penguin brain tissue specimens are thoroughly analysed and compared with the Raman spectra of the human brain as well as mouse brain specimens from the previously reported literatures^16,18,47–50^.

In the low wavenumber region (below 1000 cm^−1^), the first observable peak at ~ 547 cm^−1^ is assigned to vibrations related to amino acids (tryptophan) combined with nucleobases found in DNA and RNA (cytosine and guanine)^51^. In addition, the successive four peaks at ~ 623, 642 661, and 704 cm^−1^ are primarily attributed to the vibrations associated with different kinds of amino acids and correspondingly assigned to the C–C twisting mode of phenylalanine, C–C twisting mode of tyrosine, C–S stretching mode of cystine, and CH_2_ rocking coupled with symmetric breathing of L-tyrosine, respectively^54,56^. In the same spectral regime, the SERS spectra suggest the other noticeable peaks at ~ 719, 744, 880, 964, and 978 cm^−1^, which are tentatively assigned to the symmetric choline C–N stretch (membrane phospholipid head) coupled with ring breathing mode of DNA/RNA nucleobases adenine, CH_2_ rocking coupled with symmetric breathing of tryptophan, mitochondria, and cytochrome c, secondary amino acid proline, hydroxyapatite (PO_4_^3−^ symmetric stretching)/carotenoid/cholesterol, and C–H out of plane deformation coupled with C–C asymmetric stretching of Deoxygenated cells, cytochrome c, and porphyrin, respectively^52^. Notably, the low wavenumber spectral region of penguin brain tissues reveals the moderately intensified SERS peaks with close similarities with human brain specimens exhibiting the closely matched locations of relative Raman modes. In addition, the corresponding spectra depict the simultaneous presence of amino acids, nucleobases of DNA/RNA, nutrients, cholesterol, proteins, and porphyrin and thus exhibit sufficient relevance with endogenous fluorophores mentioned in autofluorescence spectra (Table 1)^16,18,47^.

Furthermore, we have identified and assigned the spectral features in the high wavenumber region (above 1000 cm^−1^) of penguin brain tissue’s SERS spectra. In this high wavenumber region, the selectively sharp Raman mode at ~ 1003 cm^−1^ is attributed to the symmetric C–C aromatic ring breathing mode of phenylalanine and collagen IV, and I, while the other distinct peaks at ~ 1067 and 1088 cm^−1^ are collectively assigned as C–C stretching coupled with C–C skeletal stretching, PO_2_ symmetric stretching of nucleic acid, protein, phospholipid, glycogen, and collagen IV. As a building block of the protein, phenylalanine is an essential amino acid that is required as a structural stabilizing factor in peptides and proteins. The associated development of the neurotransmitter norepinephrine is important for improving memory and helping in the signal transmission between the nerve cells of the brain^54^. Three closely spaced Raman modes at ~ 1131, 1158, and 1179 cm^−1^ are assigned to C–C stretching of lipids coupled with C–N stretching in proteins, C–C stretching of carotenoids coupled with C–N stretching of proteins, and C–H in-plane bending of hemoglobin, tyrosine, and flavin, respectively. Moreover, the minor peak present at ~ 1200 cm^−1^ corresponds to vibrations related to hydroxyproline and amino acids tyrosine, tryptophan, and phenylalanine. In addition, amide III clearly exhibits the Raman modes within the 1200–1300 cm^−1^ spectral region, so the two Raman peaks obtained at ~ 1273 and 1305 cm^−1^ are assigned to in-plane deformation of N–H, C–N stretching, and CH_3_ in-plane deformation of amide III^55^.

Furthermore, the same spectral regime (from 1350 to 1700 cm^−1^) depicts the two most prominent Raman modes corresponding to the sharply intense vibrational features of amide I, proteins, and lipids, showing the Raman modes at ~ 1448 cm^−1^ (δ(CN) bending, δ(CH)_3_ out-of-phase deformation of lipid and protein) and 1661 cm^−1^ (amide I (proteins with b-sheet conformational structures), ν(C=O), and collagen I, IV)^53^. The strong band at 1448 cm^−1^ is primarily ascertained to be the bending vibrations associated with the lipid molecules and plays a useful role in cell division, signal transduction, and growth activities to serve as the cellular energy storage system in the penguin brain tissues. However, the spectral region of 1600–1800 cm^−1^ is ascribed to the amide I associated vibrations mainly governed by the stretching of the C=O group, and its strong spectral intensity is particularly related to the proteins and polypeptides for maintaining the chemical balance in the brain. Other minor peaks are assigned at ~ 1351 cm^−1^ (CH_3−_ (C=O) tryptophan, mitochondria, cytochrome c), 1384 cm^−1^ (CH_3_ in-phase deformation, methyl symmetric bending of thymine, adenine, and guanine of DNA), 1399 cm^−1^ (CH_3_ bending due to methyl bond in the membrane), 1556 cm^−1^ (C=C tryptophan, porphyrin), and 1622 cm^−1^ (C=C stretching mode of tyrosine and tryptophan).

The analysis of penguin brain tissue using SERS enabled the extraction of valuable information regarding Raman features across the entire spectral region. The close observation visibly indicates the reasonable differentiations and similarities of the penguin brain tissues compared to the human and mouse brains^16,18,47–50^. The penguin brain tissue specimens depicted distinctly sharp Raman features; however, they are accompanied by slight spectral differences. In comparison, the penguin brain Raman spectrum critically entailed most of the spectral features associated with the spectral characteristics of mouse brain specimens with comparatively distinct spectral features at ~ 704, 1003, 1067, 1305, 1448, and 1661 cm^−1^ depicting the healthy presence of amino acids, nucleobases in DNA/RNA, lipids, nucleic acid, phenylalanine, proteins, and amides in the penguin brain tissue^48–50^. In addition, the comparatively sharp Raman bands corresponding to lipids (I@1448 cm^−1^) and amide I (I@1661 cm^−1^) compared to the human brain and mouse brain show remarkable spectral differences^16,18,47–50^. The comparative analysis evidently revealed the slightly altered relative peak positions in terms of reduced (in comparison to the human brain) and increased (in contrast to the mouse brain) intensities of Raman peaks associated with phenylalanine (I@1003 cm^−1^) vibrational mode. These marked spectral alterations are sensitively attributed to the comparative structural conformations of the constituents of the penguin brain tissue specimens. Furthermore, the low and high wavenumber regions of the SERS spectra of penguin brain tissue specimens depict reasonable consistencies with the endogenous fluorophores exhibiting autofluorescence spectra through the simultaneous presence of collagen, porphyrin, nucleic acids, etc. All the Raman and SERS spectra were background subtracted using WIRE software with the Ranishaw InVia Raman spectrometer.

## Conclusion

In this study, we focus on analysing the morphological and molecular characteristics of penguin brains to understand their functionality and sustenance in Antarctica’s extreme climate. We employed label-free optical imaging and spectroscopic techniques to investigate the neurological structure and properties inherent in penguin brains. Our approach utilizes QPI, AF, and Raman spectroscopy to identify and analyze microscopic and molecular features in penguin brain tissue. QPI provides high contrast, quantitative phase information on cellular and organelle structures, complementing histological sections stained with CV to visualize brain tissue morphology. Integrating QPI enhances our understanding of penguin brain intricacies, offering valuable insights for further study. AF spectroscopy captures spectral profiles ranging from 450 to 550 nm, offering molecular insights into NADH and FAD, while in the 420–520 nm range, it provides information on elastin collagen crosslinks, with discernible peaks of porphyrins around ~ 600 nm, etc. Raman spectroscopy reveals prominent peaks indicative of amides and protein deformation at approximately 1305 cm^−1^, 1661 cm^−1^, and 1448 cm^−1^, respectively. The study signifies that the low-wavenumber spectral region of the penguin brain tissues reveals moderately intensified SERS peaks, which show close similarities to human brain specimens. Also, the relative Raman modes are closely matched with the human brain, and the spectra provide significant biochemical insights, displaying the simultaneous presence of amino acids, nucleobases of DNA/RNA, nutrients, cholesterol, proteins, and porphyrins. These findings contribute to our understanding of the neurological characteristics of penguin brains and pave the way for the development of a digital brain atlas.

## Supplementary Information

Supplementary Information.

## Data Availability

The datasets generated during and/or analysed during the current study are available from the corresponding author on reasonable request.
