# Supplementary material for: Investigation on morphological and molecular fingerprints of penguin brain using label-free optical imaging and spectroscopic techniques
Source: Sci Rep. 2025 Feb 10;15:4923. doi: 10.1038/s41598-024-76127-0 (PMC11811042; doi:10.1038/s41598-024-76127-0)
Supplement: Supplementary file 1 — Supplementary Information. [file 41598_2024_76127_MOESM1_ESM.pdf]

Supplementary information

Investigation on morphological and molecular fingerprints of penguin brain using label-free optical imaging and spectroscopic techniques

Sunil Bhatt,[1, #, †] Ashwani Kumar Verma,[1, †] Prabir G. Dastidar,[2, $] Punit Kumar,[3] Pramila Thapa,[1] Tony George Jacob,[3] Tara Sankar Roy,[4] Soumya Iyengar,[5] Senthil Kumaran,[3] Balpreet Singh Ahluwalia,[6] and Dalip Singh Mehta[1, *]

[1]*Bio-photonics and Green-photonics Laboratory, Department of Physics, Indian Institute of Technology Delhi, Hauz-Khas, New Delhi 110016, India.*

[2] *Polar Sciences division, Ministry of Earth Sciences, New Delhi 110003.*

[3]*Department of Anatomy, All India Institute of Medical Sciences (AIIMS), Delhi, India.*

[4]*Department of Anatomy, North Delhi Municipal Corporation Medical College and Hindu Rao Hospital, Delhi, India.*

[5]*National Brain Research Centre, Manesar.*

[6]*Department of Physics and Technology, UiT The Arctic University of Norway, Norway.*

[*]mehtads@physics.iitd.ac.in, [#]sunilbhatt.619@gmaail.com, [$]prabirgd11@gmail.com

[†]Contributed equally to this work

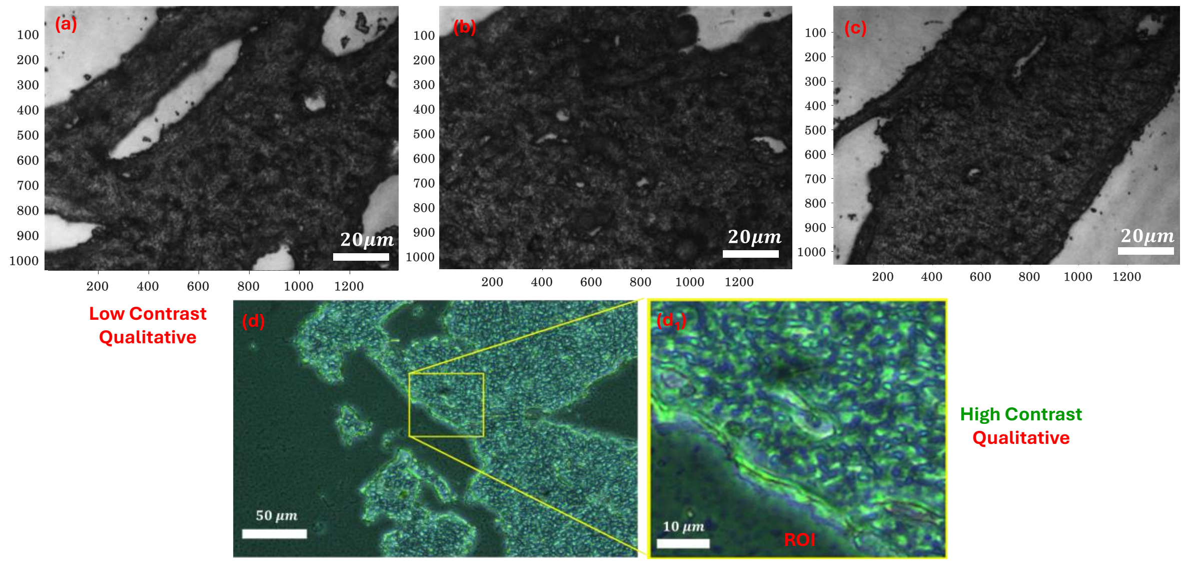

Fig. S1 Bright field (BF) and phase contrast (PC) microscopy of penguin brain sample. (a-c) are the BF images of the brain sample with low contrast and no quantitative information. (d) is the PC image, and (d_1) is the zoomed region of interest (ROI), which visualizes the high-contrast image of different cellular structures.

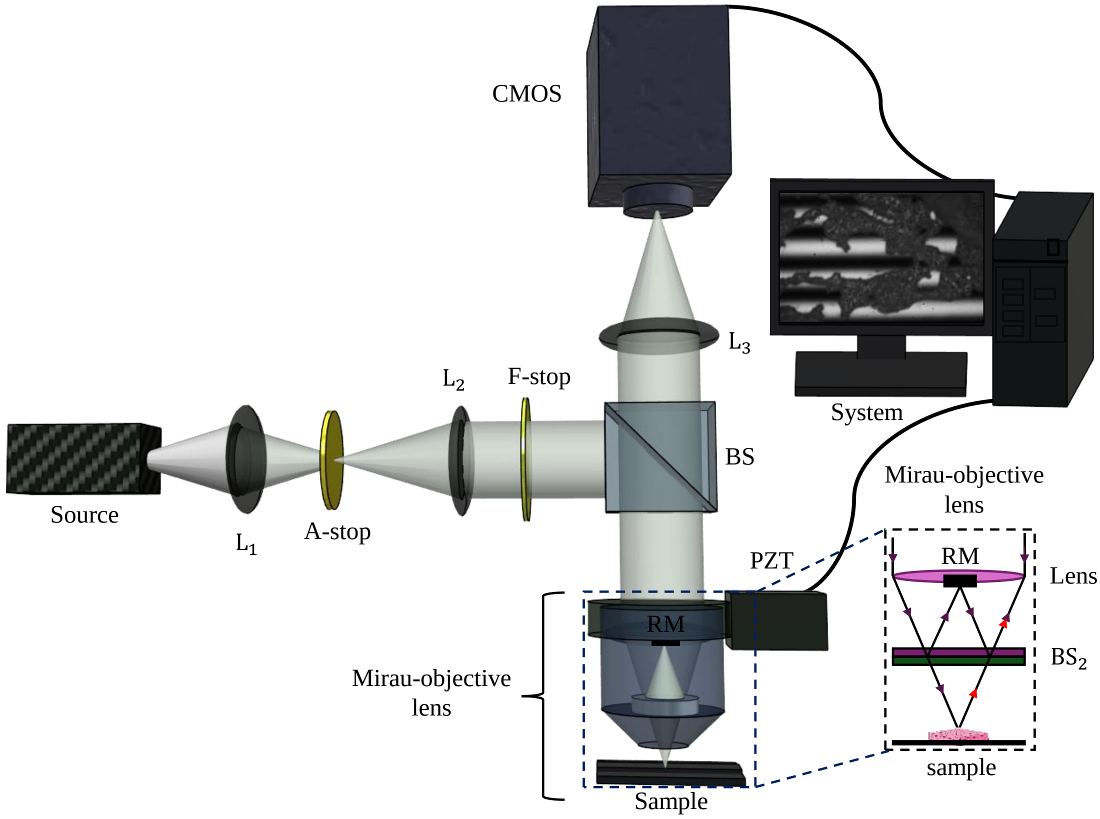

Fig. S2 Illustrating the schematic diagram of white light phase-shifting interference microscopy system for QPI of penguin brain sample. $L_{1\text{-}3}$: lenses, A-stop: aperture-stop, F-stop: field-stop BS: beam splitter, RM: reference mirror, PZT: piezoelectric transducer, CMOS: complementary metal oxide semiconductor.

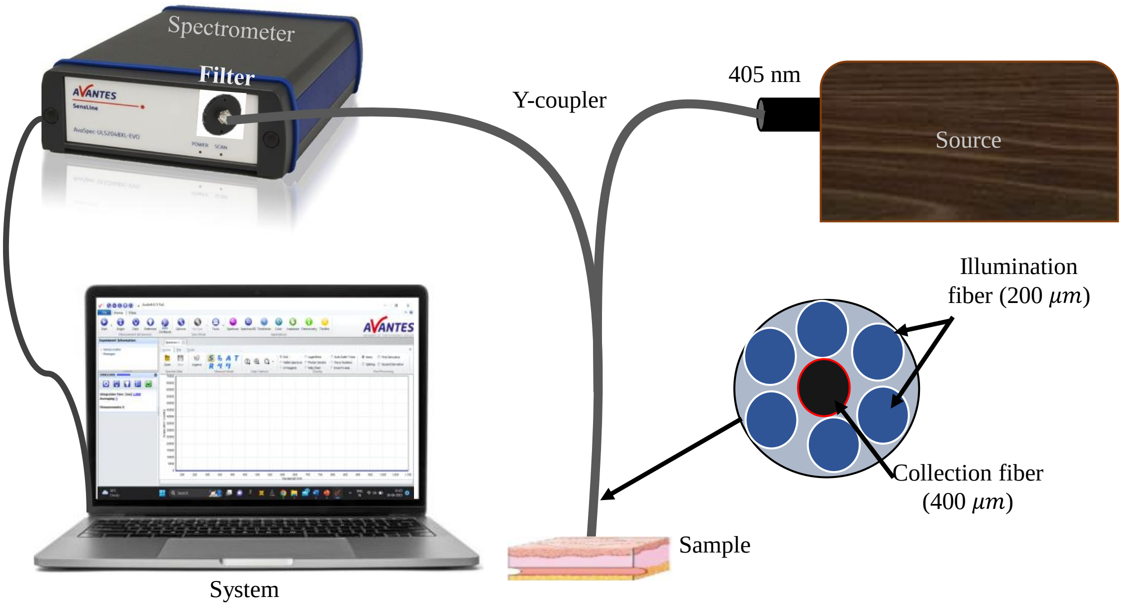

Fig. S3 Illustrating the autofluorescence spectroscopy system for the autofluorescence spectrum recording of penguin brain sample using two different excitation wavelength light sources 405nm.

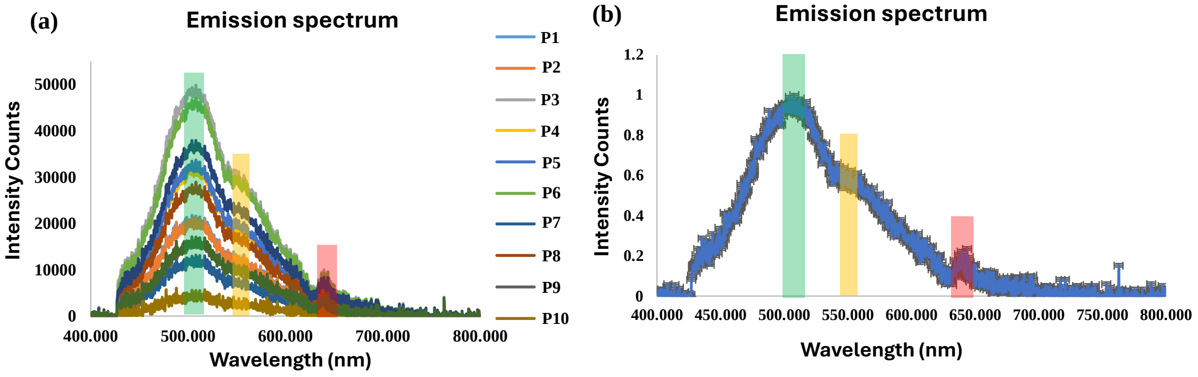

Fig. S4 Autofluorescence spectroscopy of another penguin brain at excitation wavelength 405 nm (a) AF emission spectrum of a total of 11 positions of the penguin brain with intensity variation. (b) AF spectra of the average of (a) with the standard deviation. The highlighted, green-shaded region in the spectra consistently manifests a peak around ~510 nm, corresponding to NADH and FAD crosslinks. The highlighted, red-shaded region within the spectra depicts peak maxima approximately at ~656 nm, indicative of porphyrins. The highlighted, yellow-shaded region in a range of 550 nm to 600 nm indicates lipofuscins.

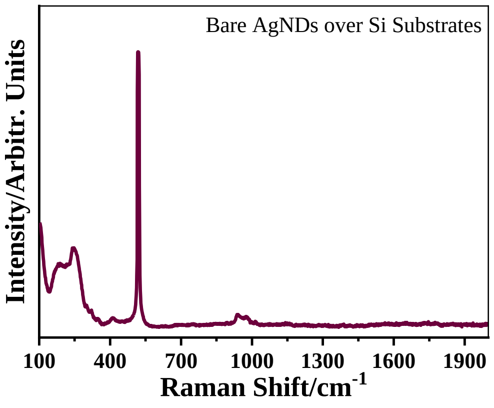

Fig. S5 Raman spectrum of bare AgNDs deposited over piranha-cleaned silicon substrates under 785 nm laser excitation wavelength.

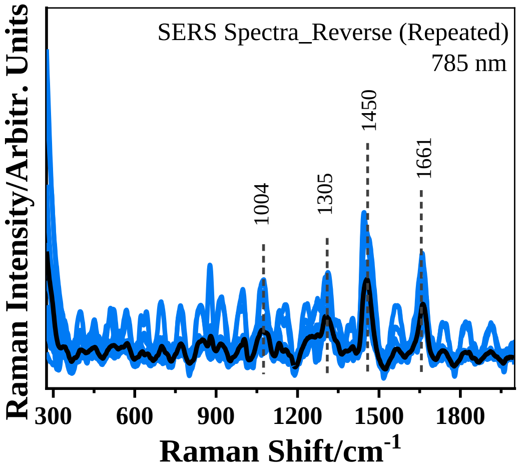

Fig. S6 The collected average SERS spectra of another penguin brain at 10 different positions by reversely drop-coating the colloidal AgNDs solution on the top of the tissue specimens under NIR (785 nm) excitation wavelength.

SUPPLEMENTARY METHODS

LABEL-FREE OPTICAL MICROSCOPIC AND SPECTROSCOPIC STUDY OF PENGUIN BRAIN

S1. EXPERIMENTAL SETUP USED FOR QUANTITATIVE PHASE IMAGING

The experimental setup of a white light phase-shifting interference microscopy (WL-SPIM) system for QPI of penguin brain sample is shown in Fig. S2. A white light is used as a source to illuminate the sample, and two lens combinations L_1 and L_2 are used to collect the incident beam from the source and collimate the beam towards the Mirau objective lens using a beam splitter BS. An aperture-stop (A-stop) and a field-stop (F-stop) are used to control the aperture and field of view of the incident beam, respectively, ultimately, control the spatial coherence, which results in control contrast of the interferogram in WL-PSIM [1]. The incident beam in the Mirau objective lens focused on the sample and the reference mirror (RM) inside the objective lens, and the back-reflected light from the sample and RM interfered at the BS_2 plane, which further projected towards the camera plane using the tube lens L_3. A piezoelectric transducer (PZT) is used to introduce the equal temporal phase shift between the multiple data frames to perform the final phase reconstruction using the phase-shifting algorithm. Using PZT, we recorded five phase-shifted interferograms, which were further used to reconstruct the sample phase using the five phase-shifting algorithms. The blue rectangular box represented the ray diagram of the Mirau objective lens, where the violet and red arrows represent the back-reflected light from the RM and samples that interfered at the BS_2 plane, respectively.

PRINCIPLE OF QUANTITATIVE PHASE IMAGING

The fundamental principle of QPI is to measure the phase shift ($\Delta\phi$) governed by the cell's thickness (d) and refractive index (n) variation throughout the sample [1-3]. In QPI, the phase shift is determined in terms of the path length difference introduced by the cells between the two arms of the interferometric system [2]. The schematic diagram of WL-PSIM is shown in Fig S2. The basic idea behind phase-shifting interferometry (PSI) is to introduce systematic phase shifts between reference and sample fields [1]. In WL-PSIM, the broadband light sources such as white light, superluminescent diodes, LEDs, etc., are used to obtain the interference pattern when the optical path length $\leq L_c$ (coherence length of the light source). However, in such a technique, high fringe density over the camera's whole field of view (FOV) cannot be achieved due to poor coherence length (coherence length of white light halogen lamp l_c ~1–2µm) of a broadband source. The piezoelectric transducer (PZT) is used to control the phase shift between the data frames. Thus, we require multiple phase-shifted interferograms for the final phase reconstruction of the samples, which contains sample thickness and refractive index information. In WLPSIM, the intensity $I(x, y, \lambda_j)$ distribution of an interferogram recorded by a camera is given by [1,4];

$$I\left(x,y,\lambda_j\right) = I_s\left(x,y,\lambda_j\right) + I_r\left(x,y,\lambda_j\right) + 2\sqrt{I_s\left(x,y,\lambda_j\right)I_r\left(x,y,\lambda_j\right)}\,\gamma_{12}(\tau)\cos\left[\phi\left(x,y,\lambda_j\right) + \delta_i(t)\right] \quad (1)$$

where j corresponds to a different wavelength inside the white light source, $I_s(x,y,\lambda_j)$, $I_r(x,y,\lambda_j)$ are the intensity of light from sample and reference beams, $\gamma_{12}(\tau)$ is the degree of coherence, $\phi(x,y,\lambda_j)$ is a 2D phase map, and $\delta(t)$ is the controlled phase shift added purposely between the reference and sample field. To extract the phase $\phi(x,y,\lambda_j)$ of the object, five phase-shifted interferograms, with a controlled phase-shift δ_i (t) between reference and sample field is needed [4,5]. $\delta_i(t) = -2\alpha, -\alpha, 0, \alpha, 2\alpha$; where i = 1, 2, 3, 4, 5, 'α' is the linear phase-shift between frames and 'i' corresponds to a single phase-shifted interferogram. By solving five equations for $\alpha = \frac{\pi}{2}$, the final required expression to extract the phase of the object using five-step phase-shifting algorithm [1,4].

$$\Delta\phi\left(x,y,\lambda_j\right) = \tan^{-1}\left[\frac{2\left(I_4\left(x,y,\lambda_j\right) - I_2\left(x,y,\lambda_j\right)\right)}{I_1\left(x,y,\lambda_j\right) - 2I_3\left(x,y,\lambda_j\right) + I_5\left(x,y,\lambda_j\right)}\right] \quad (2)$$

The phase information $\Delta\phi(x, y, \lambda_i)$ of the sample is given by the following expression [3,6,7];

$$\Delta\phi(x, y, \lambda_j) = \frac{2\pi t(x, y)}{\lambda_j}[n_s(\lambda_j) - n_m(\lambda_j)] \tag{3}$$

where n_s, n_m are the refractive index of the sample and surrounding medium, respectively. By using equations (2) and (3), one can extract the coupled information of the refractive index and thickness of the samples.

S2. EXPERIMENTAL SPECIFICATION USED DURING AUTOFLUORESCENCE SPECTROSCOPY

The illustration of the experimental setup for an autofluorescence spectroscopy system is shown in Fig. S3. The Spectroscopic system contains a spectrometer (Avantes), a Y-coupler (Avantes), an excitation light source having a central wavelength of 405 nm (laser pointer), a filter (long pass filter: 420 nm), and a system for recording and analysis. In the system, the Y-coupler is connected to the spectrometer with a filter assembly to separate out the excitation light, and another end of the coupler is connected to the light source. The excitation light source excites the sample through illumination fibers, collects an autofluorescence signal through the collecting end, and filters out the excitation light at the spectrometer end. A spectrometer is connected to the system, which records the autofluorescence spectra and performs the analysis. By placing the long pass filter of 420nm in the detection arm for excitation light source 405nm, we recorded autofluorescence spectra with multiple peaks that correspond to the biomarkers of the brain sample.

PRINCIPLE OF AUTOFLUORESCENCE SPECTROSCOPY

Biomolecules found within the human body, such as nicotinamide adenine dinucleotide (NADH), flavin adenine dinucleotide (FAD), collagen, porphyrins, etc., possess inherent fluorescence properties, mean by emit light when excited by an appropriate energy source[8-12]. This phenomenon, known as autofluorescence, can be measured and analyzed to obtain autofluorescence emission spectra. The controlled interaction of light with tissues plays a crucial role in studying biomolecules present in different tissues, particularly when examining subtle biological changes, as it provides insights into molecular energy levels [9,10]. Autofluorescence spectra recording enables the identification of vibrational and electronic changes in the molecules, as indicated by variations in the full-width half maximum (FWHM) of the spectra[10]. According to the Frank-Condon principle, changes in FWHM suggest molecular transitions occurring between energy levels that possess the same vibronic phase value. Consequently, even slight alterations in the biological state can be reflected in changes in FWHM. Moreover, deviations in the peak emission wavelength of intrinsic fluorophores may indicate energy transfer or reabsorption processes taking place within the molecules[10].

S3. EXPERIMENTAL SPECIFICATION USED DURING RAMAN SPECTROSCOPY

The plasmon response and the morphological analysis of the as-synthesized AgNDs were performed by UV-Vis spectrophotometer (Perkin Elmer Lambda 35) and field emission scanning electron microscopy (FESEM) (JEOL JSM-7800F Prime), respectively. All the normal Raman and SERS spectra measurements of the thick penguin brain tissue specimens were acquired by Renishaw, in-Via micro-Raman spectrometer equipped with a Leica optical microscope, illuminated by a 785nm diode laser while maintaining similar experimental conditions. In order to clearly identify the molecular fingerprints of the penguin brain, the multiple spectra of the tissue specimens were collected from the randomly selected locations after the drop-coating of dendritic-shaped silver nanostructures. Under the laser exposure, the sample photoreaction and heating issues were prevented by focusing the tissue specimens with low (~ 10 mW) laser power levels employing a 10× microscope objective, and all the Raman spectra were acquired for 10s integration time for two consecutive accumulations. Both penguin brain tissue specimens were investigated by collecting the SERS spectra from the 15 (penguin brain 1) and 10 (penguin brain 2) different locations and then averaged out. All the Raman and SERS spectra were background subtracted using WIRE software with the Renishaw InVia Raman spectrometer.

PRINCIPLE OF SURFACE-ENHANCED RAMAN SPECTROSCOPY

Raman scattering is an inherently feeble phenomenon, as only approximately 1 in ~10^6-10^{10} photons are scattered inelastically, thus limiting its practicability for sensitive analyte identification applications [13]. However, ultra-sensitive fingerprint identification of chemical and biological analytes at trace or single-molecular levels is highly desirable for both scientific and technological advancements. The inherently weak Raman signal can be significantly enhanced through the resonance excitation of analytes via illuminating with the laser energy close to the electronic transition energy of analytes, known as the Resonance Raman (RR) effect [14]. Recently, plasmonic metal nanoparticles (MNPs) have been extensively investigated for the SERS-based detection of different chemicals and biomolecules targeting disease diagnosis, medical analysis, environmental and food safety [15,16]. SERS offers highly sensitive, specific, reproducible, and multiplexed identification of a wide range of analyte molecules from the interfering background species, such as from extracellular fluids and tissue specimens [17-19]. The SERS sensitivity is primarily controlled by the localization of electromagnetic fields at the sharp features and within inter-particle nano-sized gaps due to the collective oscillation of the conduction electrons on the metallic features caused by light irradiation [20]. However, the attachment or interaction of the adsorbing analyte with the metallic surfaces can induce the charge transfer at the molecule–metal interface and critically improve the overall intensity enhancement factors (EFs) [21]. The generation of structural anisotropy in the nanoparticle shape produces multiple hot-spots in a single nanostructure without the salt-addition-induced nanoparticle aggregation processes and now emerged as an efficient tool to scale up the sensitivity of the SERS substrates. Importantly, MNPs with highly branched morphologies generate the three-dimensional (3D) SERS 'hot-spots' and accordingly the strong plasmonic response [22-24]. Owing to the unique structural properties of branched nanostructures, such as AgNDs provide a high surface area for the interaction of analytes along with intriguing sharp micro/nano-sized features and gaps for the efficient concentration of electric fields and correspondingly exhibit superior SERS performance [23,24].

References

1 Bhatt, S., Butola, A., Kanade, S. R., Kumar, A. & Mehta, D. S. High-resolution single-shot phase-shifting interference microscopy using deep neural network for quantitative phase imaging of biological samples. *Journal of Biophotonics* **14**, e202000473 (2021).

2 Popescu, G. *Quantitative phase imaging of cells and tissues*. (McGraw-Hill Education, 2011).

3 Butola, A. *et al.* High space-bandwidth in quantitative phase imaging using partially spatially coherent digital holographic microscopy and a deep neural network. *Optics Express* **28**, 36229-36244 (2020).

4 Hariharan, P., Oreb, B. F. & Eiju, T. Digital phase-shifting interferometry: a simple error-compensating phase calculation algorithm. *Appl. Opt.* **26**, 2504-2506 (1987).

5 Schreiber, H. & Bruning, J. H. Phase shifting interferometry. *Optical shop testing*, 547-666 (2007).

6 Machikhin, A., Polschikova, O., Ramazanova, A. & Pozhar, V. Multi-spectral quantitative phase imaging based on filtration of light via ultrasonic wave. *Journal of Optics* **19**, 075301 (2017).

7 Bhatt, S. *et al.* Single-shot multispectral quantitative phase imaging of biological samples using deep learning. *Appl. Opt.* **62**, 3989-3999 (2023).

8 Chorvat Jr, D. & Chorvatova, A. Multi-wavelength fluorescence lifetime spectroscopy: a new approach to the study of endogenous fluorescence in living cells and tissues. *Laser Physics Letters* **6**, 175-193 (2009).

9 Thapa, P. *et al.* Multimodal fluorescence imaging and spectroscopic techniques for oral cancer screening: a real-time approach. *Methods Applications in Fluorescence* **11**, 045008 (2023).

10 Lakowicz, J. R. *Principles of fluorescence spectroscopy*. (Springer, 2006).

11 Ramanujam, N. Fluorescence spectroscopy in vivo. *Encyclopedia of analytical chemistry* **1**, 20-56 (2000).

12 Richards-Kortum, R. & Sevick-Muraca, E. Quantitative optical spectroscopy for tissue diagnosis. *Annual review of physical chemistry* **47**, 555-606 (1996).

13 Le Ru, E. & Etchegoin, P. *Principles of Surface-Enhanced Raman Spectroscopy: and related plasmonic effects*. (Elsevier, 2008).

14 Robert, B. Resonance raman spectroscopy. *Photosynthesis research* **101**, 147-155 (2009).

15 Pilot, R. *et al.* A review on surface-enhanced Raman scattering. *Biosensors* **9**, 57 (2019).
16 Reguera, J., Langer, J., de Aberasturi, D. J. & Liz-Marzán, L. M. Anisotropic metal nanoparticles for surface-enhanced Raman Scattering. *Colloidal Synthesis of Plasmonic Nanometals*, 713-754 (2020).
17 Alvarez-Puebla, R. A., Li, J.-F. & Ling, X. Y. Introduction to advances in plasmonics and its applications. *Nanoscale* **13**, 5935-5936 (2021).
18 Deriu, C., Thakur, S., Tammaro, O. & Fabris, L. Challenges and opportunities for SERS in the infrared: materials and methods. *Nanoscale Advances* (2023).
19 Sharma, B., Frontiera, R. R., Henry, A.-I., Ringe, E. & Van Duyne, R. P. SERS: Materials, applications, and the future. *Materials today* **15**, 16-25 (2012).
20 Ding, S.-Y., You, E.-M., Tian, Z.-Q. & Moskovits, M. Electromagnetic theories of surface-enhanced Raman spectroscopy. *Chemical Society Reviews* **46**, 4042-4076 (2017).
21 Valley, N., Greeneltch, N., Van Duyne, R. P. & Schatz, G. C. A look at the origin and magnitude of the chemical contribution to the enhancement mechanism of surface-enhanced Raman spectroscopy (SERS): Theory and experiment. *The Journal of Physical Chemistry Letters* **4**, 2599-2604 (2013).
22 Garcia-Leis, A., Garcia-Ramos, J. V. & Sanchez-Cortes, S. Silver nanostars with high SERS performance. *The Journal of Physical Chemistry C* **117**, 7791-7795 (2013).
23 Verma, A. K. & Soni, R. Silver nanodendrites for ultralow detection of thiram based on surface-enhanced Raman spectroscopy. *Nanotechnology* **30**, 385502 (2019).
24 Verma, A. K. & Soni, R. K. Ultrasensitive surface-enhanced Raman spectroscopy detection of explosive molecules with multibranched silver nanostructures. *Journal of Raman Spectroscopy* **53**, 694-708 (2022).
